# Supplementary material for: BMP7 increases protein synthesis in SW1353 cells and determines rRNA levels in a NKX3-2-dependent manner
Source: PLoS One. 2022 Feb 9;17(2):e0263430. doi: 10.1371/journal.pone.0263430 (PMC8827423; doi:10.1371/journal.pone.0263430)
Supplement: S1 File — (PDF) [file pone.0263430.s004.pdf]

SUPPORTING INFORMATION TO:

BMP7 increases protein synthesis in SW1353 cells and determines rRNA levels in a NKX3-2-dependent manner

Ellen G.J. Ripmeester[1], Tim J.M. Welting[1,2], Guus G.H. van den Akker[1], Don A.M. Surtel[1], Jessica S.J. Steijns[1], Andy Cremers[1], Lodewijk W. van Rhijn[1,2], Marjolein M.J. Caron[1*]

[1] Laboratory for Experimental Orthopedics, Department of Orthopedic Surgery, Maastricht University, Maastricht, the Netherlands

[2] Laboratory for Experimental Orthopedics, Department of Orthopedic Surgery, Maastricht University Medical Center, Maastricht, the Netherlands

* Corresponding author

E-mail: marjolein.caron@maastrichtuniversity.nl

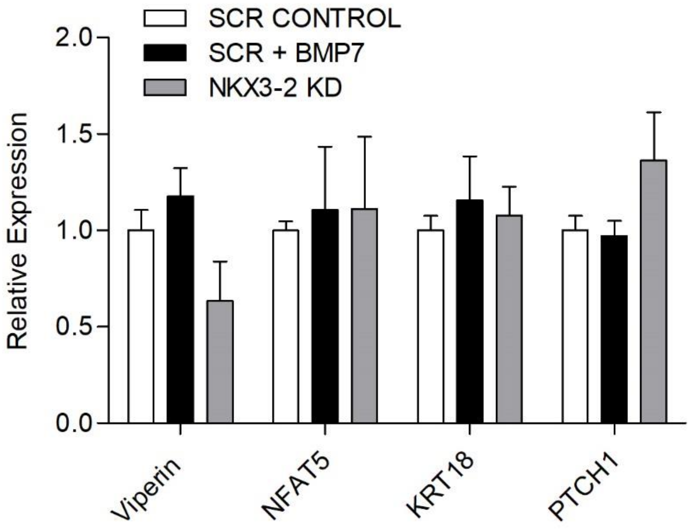

S1 Fig: Unrelated/unaffected genes which are not controlled by the BMP7-NKX3-2- rRNA axis. A/B. SW1353 cells were transfected with either a scrambled (SCR) or NKX3-2 (NKX3-2 KD) siRNA duplex (100nM) and exposed to BMP7 (1nM) for 24 hours after which expression levels of Viperin, NFAT5, KRT18 or PTCH1 were determined using RT-qPCR analysis. Data were normalized to cyclophilin expression and set relative to the SCR control condition (n=3 samples per condition). Statistical significance was determined using a two-tailed unpaired Student's t-tests, an no significant changes for each gene between conditions was observed. Bars show the mean ±SEM. * P<0.05, ** P<0.01, *** P<0.001.

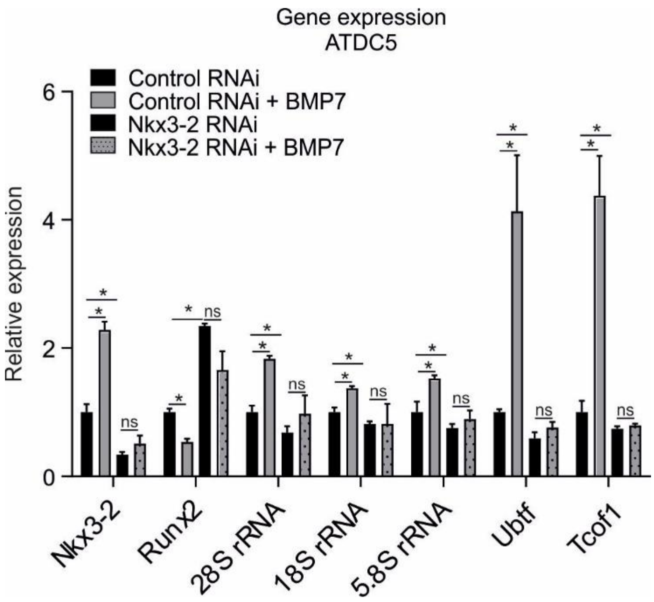

S2 Fig: BMP7-induced rRNA levels are NKX3-2 dependent in ATDC5 cells

ATDC5 cells (6.400 cells/cm^2) were differentiated in the chondrogenic lineage for 6 days to acquire a chondrocyte phenotype and then transfected (according to the manufacturer's protocol, using HiPerfect, Qiagen) with either a scrambled (Control RNAi (Eurogentec)) or Nkx3-2 (Nkx3-2 RNAi) siRNA duplex (100nM; sense: 5'-CAGAGACGCAAGUGAAGAUTT-3', anti-sense: 5'-AUCUUCACUUGCGUCU CUGTT-3') and exposed to BMP7 (1nM) for 24 hours after which expression levels of Nkx3-2, Runx2, 18S rRNA, 5.8S rRNA, 28S rRNA, Ubtf and Tcof1 were measured by RT-qPCR. Data were normalized to cyclophilin expression and set relative to the control condition (n=3 samples per condition). Differentiation medium for ATDC5 consisted of Dulbecco's minimal essential medium (DMEM)/F12 (Invitrogen), 5% fetal calf serum (FCS) (Sigma-Aldrich), 1% antibiotic/antimycotic (Invitrogen) and 1% NEAA (non-essential amino acids; Invitrogen), 10mg/ml insulin (Sigma-Aldrich),10mg/ml transferrin (Roche) and 30 nM sodium selenite (Sigma-Aldrich). Statistical significance was determined using a two-tailed unpaired Student's t-tests. Bars show the mean ±SEM. * P<0.05, ** P<0.01, *** P<0.001. ns= not significant.

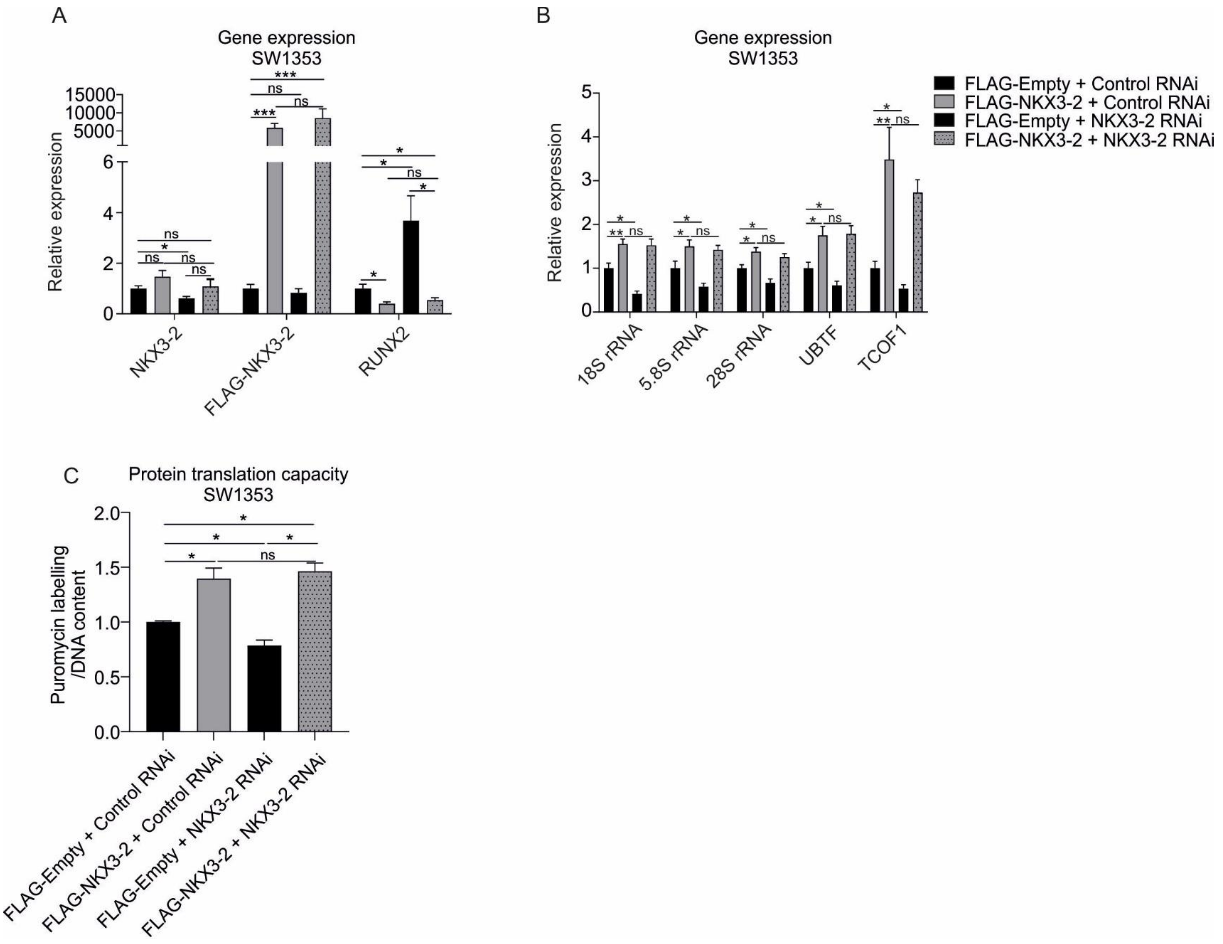

S3 Fig: NKX3-2 overexpression increases rRNA levels and is associated with increased translational capacity

A-B. NKX3-2 was overexpressed by transient transfection of a codon usage optimized FLAG-NKX3-2 vector into SW1353 cells and FLAG-empty vector was used as a negative control. The next day, these cells were transfected with either a scrambled (Control RNAi) or NKX3-2 (NKX3-2 RNAi) siRNA duplex (100nM) and after 24 hours cells were harvested. Expression of mRNA for the indicated genes was determined by real-time RT-qPCR. Data were normalized to cyclophilin expression and set relative to the control condition (A-B: n=3 samples per condition). **C**. Translational capacity was determined and Puromycilation data were normalized to DNA content and calculated relative to the control condition (n=5 samples per condition). Statistical significance was determined using unpaired two-tailed Student's t-tests. Bars show the mean ±SEM. * P<0.05, ** P<0.01, *** P<0.001 versus control conditions. ns = not significant.
