# Supplementary material for: BMP7 increases protein synthesis in SW1353 cells and determines rRNA levels in a NKX3-2-dependent manner
Source: PLoS One. 2022 Feb 9;17(2):e0263430. doi: 10.1371/journal.pone.0263430 (PMC8827423; doi:10.1371/journal.pone.0263430)
Supplement: Attachment — Submitted filename: Ripmeester et al. Response to reviewers 15112021.pdf [file pone.0263430.s006.pdf]

PONE-D-20-37112R1
BMP7 increases protein synthesis in SW1353 cells and determines rRNA levels in a NKX3-2-dependent manner
PLOS ONE

Dear Dr. Caron,

Thank you for submitting your revised manuscript to PLOS ONE. After careful consideration, we feel that it has merit but does not fully meet PLOS ONE's publication criteria as it currently stands. Therefore, we invite you to submit a revised version of the manuscript that addresses the points raised during the review process.
One of the reviewers expressed residual concerns that some of the additional work that was recommended, while logistically very difficult under COVID conditions, would still add value to this work. From an editorial perspective, we have rendered an initial decision based on the reviews of only one reviewer to expedite a decision in light of the fact that many suitable reviewers were not available to assess your work. Since this reduced the number of points that needed to be addressed, it is perhaps not unreasonable for you to reconsider what could be done to satisfy the recommendations of this single reviewer.
Upon personal review of this work, I appreciate the importance of your work. However, it is objectively evident that your paper, even with the supplementary figures you provided, contains a rather minimal amount of data documented in Figures 1 and 3, beyond a simple two component bar graph in Figure 2. Papers for PLOS One typically present a larger volume of high quality work.
Moving forward, I recommend that you follow the advice of the reviewer and let this paper mature and include a bit more data at an extended deadline to allow you to address the original critique. In addition, I believe that the Supplementary Figures should be incorporated in the main text because they present important context for the main findings. With the number of figures even in the revised paper you are not reaching a maximal figure limit yet. We trust that you will be able to make additional modifications, but feel free to contact us by email if you have questions regarding what you could realistically do and not do with an extended deadline.
Please submit your revised manuscript by May 13 2021 11:59PM. If you will need more time than this to complete your revisions, please reply to this message or contact the journal office at plosone@plos.org. When you're ready to submit your revision, log on to https://www.editorialmanager.com/pone/ and select the 'Submissions Needing Revision' folder to locate your manuscript file.

Please include the following items when submitting your revised manuscript:

- A rebuttal letter that responds to each point raised by the academic editor and reviewer(s). You should upload this letter as a separate file labeled 'Response to Reviewers'.
- A marked-up copy of your manuscript that highlights changes made to the original version. You should upload this as a separate file labeled 'Revised Manuscript with Track Changes'.
- An unmarked version of your revised paper without tracked changes. You should upload this as a separate file labeled 'Manuscript'.

If you would like to make changes to your financial disclosure, please include your updated statement in your cover letter. Guidelines for resubmitting your figure files are available below the reviewer comments at the end of this letter.

If applicable, we recommend that you deposit your laboratory protocols in protocols.io to enhance the reproducibility of your results. Protocols.io assigns your protocol its own identifier (DOI) so that it can be cited independently in the future. For instructions see: http://journals.plos.org/plosone/s/submission-guidelines#loc-laboratory-protocols. Additionally, PLOS ONE offers an option for publishing peer-reviewed Lab Protocol articles, which describe protocols hosted on protocols.io. Read more information on sharing protocols at https://plos.org/protocols?utm_medium=editorial-email&utm_source=authorletters&utm_campaign=protocols.

We look forward to receiving your revised manuscript.

Kind regards,

Andre van Wijnen
Academic Editor
PLOS ONE

- Authors' response: We thank the Academic Editor for carefully rereading our manuscript and the generous opportunity and time to revise our manuscript based on comments from the Academic Editor and the Reviewer.
- To meet the Editors' request regarding the rather minimal amount of data presented in the Figures, we repeated the majority of the experiments in human primary chondrocytes (3 independent donors) and obtained similar results. These data are now added to the manuscript (Figure 1B, 1D, 2C, 3G and 4C). Moreover, we moved the NKX3-2 overexpression gene expression data that was presented in S2 Fig to the main manuscript (Figure 4), repeated the experiment in human primary chondrocytes and added translational capacity data for SW1353 cells. In addition to the presented rRNA promoter data presented in Figure 2, we now measured gene expression of UBTF and TCOF1, two factors critically involved in the transcription of the 47S precursor rRNA (Figure 2, 3 and 4). We find that this additional work adds value to the original presented work and now adheres to the volume and quality of work for publication in PLOS One.

Comments to the Author

1. If the authors have adequately addressed your comments raised in a previous round of review and you feel that this manuscript is now acceptable for publication, you may indicate that here to bypass the "Comments to the Author" section, enter your conflict of interest statement in the "Confidential to Editor" section, and submit your "Accept" recommendation.

Reviewer #1: (No Response)

---

2. Is the manuscript technically sound, and do the data support the conclusions?

The manuscript must describe a technically sound piece of scientific research with data that supports the conclusions. Experiments must have been conducted rigorously, with appropriate controls, replication, and sample sizes. The conclusions must be drawn appropriately based on the data presented.

Reviewer #1: Partly

---

3. Has the statistical analysis been performed appropriately and rigorously?

Reviewer #1: Yes

---

4. Have the authors made all data underlying the findings in their manuscript fully available?

The PLOS Data policy requires authors to make all data underlying the findings described in their manuscript fully available without restriction, with rare exception (please refer to the Data Availability Statement in the manuscript PDF file). The data should be provided as part of the manuscript or its supporting information, or deposited to a public repository. For example, in addition to summary statistics, the data points behind means, medians and variance measures should be available. If there are restrictions on publicly sharing data—e.g. participant privacy or use of data from a third party—those must be specified.

Reviewer #1: Yes

---

5. Is the manuscript presented in an intelligible fashion and written in standard English?

PLOS ONE does not copyedit accepted manuscripts, so the language in submitted articles must be clear, correct, and unambiguous. Any typographical or grammatical errors should be corrected at revision, so please note any specific errors here.

Reviewer #1: Yes

---

6. Review Comments to the Author

Please use the space provided to explain your answers to the questions above. You may also include additional comments for the author, including concerns about dual publication, research ethics, or publication ethics. (Please upload your review as an attachment if it exceeds 20,000 characters)

Reviewer #1: The authors have responded in a fashion that IF they carried through to complete the studies requested might address earlier critiques. However, due to COVID19 related research restrictions they are unable to complete the necessary experiments. While this is regrettable, the inability to generate this data does not reduce its importance. I would suggest that the authors be permitted an extension until such a time as research activity is restored and these studies can be completed.

- Authors' response: We thank the Reviewer for carefully rereading our manuscript and we will address the remaining issue below.

The Reviewers previous comment to our manuscript that we could not address due to COVID19 was: *Major: The siRNA studies in Figure 3 lack necessary specificity controls.*

These include; (1) the failure to use more than one NKX3-2 targeted siRNA duplex (2-3 distinct siRNAs should be used to reduce chance of off-target effects), (2) The authors should include a rescue experiment, by re-expression of a NKX3-2 mutant that evades riRNA silencing.

- Authors' response: The siRNA against NKX3-2 in this manuscript is the same as the siRNA we previously published [1], were we showed more confirmation that knockdown of NKX3-2 by this siRNA resulted in specific effects on NKX3-2 target genes. We now ordered and tested two new siRNA duplexes for human NKX3-2, but these did not result in successful knockdown of NKX3-2. As a second approach to try to answer the Reviewers request, we designed an siRNA duplex for mouse NKX3-2 and obtained a successful knockdown and similar results regarding rRNA expression in the murine ATDC5 cell line that was pre-differentiated for 5 days to obtain a chondrocyte phenotype. See figure below, these data are now added to the manuscript as Supplemental Figure 2. In addition to show more confidence in our NKX3-2 siRNA data generated in the SW1353 cells, we repeated the majority of the experiments in human primary chondrocytes (3 independent donors) using the same human NKX3-2 siRNA duplex and obtained similar results. These data are now added to the manuscript (Figure 3G and 4C).

To meet the Reviewer's second request for specificity control, we performed the requested experiment using the NKX3-2 siRNA and re-expression of NKX3-2 using a FLAG-NKX3-2 expression vector with optimized codon-usage that evades the NKX3-2 siRNA-mediated silencing. Overexpression of NKX3-2 by transfecting SW1353 cells or human primary chondrocytes (n=3 independent donors) with the FLAG-NKX3-2 vector [1] resulted in a significantly decreased expression of RUNX2 accompanied by an increased expression of rRNAs and accompanying translational capacity in these cells. These data are reciprocal to the NKX3-2 knockdown (also presented in Figure 3) and in line with the BMP7-mediated increase in rRNA expression and translation capacity from Figure 1. These effects could not be significantly altered by the NKX3-2 siRNA (see Figure below). We hope that these data provide ample additional insight for the Reviewer on the role of NKX3-2 in chondrocyte rRNA expression and translational capacity. The NKX3-2 overexpression data is now included in the main manuscript as Figure 4 and the whole figure as presented below in the Supplemental files accompanying this manuscript.

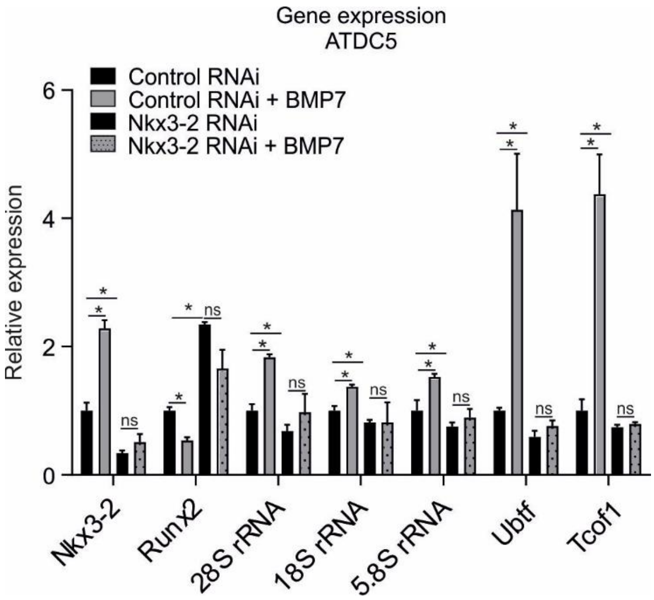

S2 Fig: BMP7-induced rRNA levels are NKX3-2 dependent in ATDC5 cells

ATDC5 cells (6.400 cells/cm^2) were differentiated in the chondrogenic lineage for 6 days to acquire a chondrocyte phenotype and then transfected (according to the manufacturer's protocol, using HiPerfect, Qiagen) with either a scrambled (Control RNAi (Eurogentec)) or Nkx3-2 (Nkx3-2 RNAi) siRNA duplex (100nM; sense: 5'-CAGAGACGCAAGUGAAGAUTT-3', anti-sense: 5'-AUCUUCACUUGCGUCUCUGTT-3') and exposed to BMP7 (1nM) for 24 hours after which expression levels of Nkx3-2, Runx2, 18S rRNA, 5.8S rRNA, 28S rRNA, Ubtf and Tcof1 were measured by RT-qPCR. Data were normalized to cyclophilin expression and set relative to the control condition (n=3 samples per condition). Differentiation medium for ATDC5 consisted of Dulbecco's minimal essential medium (DMEM)/F12 (Invitrogen), 5% fetal calf serum (FCS) (Sigma-Aldrich), 1% antibiotic/antimycotic (Invitrogen) and 1% NEAA (non-essential amino acids; Invitrogen), 10mg/ml insulin (Sigma-Aldrich),10mg/ml transferrin (Roche) and 30 nM sodium selenite (Sigma-Aldrich). Statistical significance was determined using a two-tailed unpaired Student's t-tests. Bars show the mean ±SEM. * P<0.05, ** P<0.01, *** P<0.001. ns= not significant.

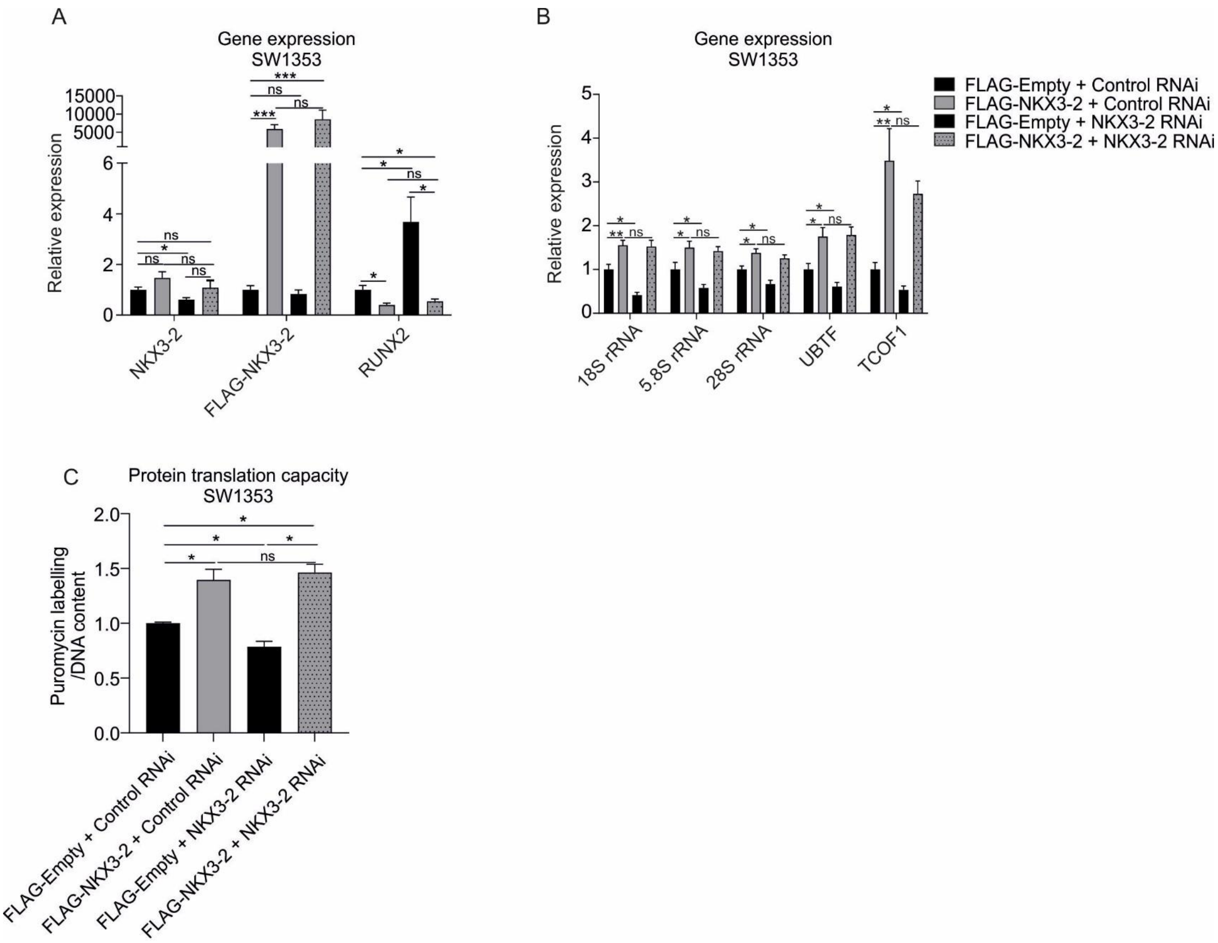

S3 Fig: NKX3-2 overexpression increases rRNA levels and is associated with increased translational capacity

A-B. NKX3-2 was overexpressed by transient transfection of a codon usage optimized FLAG-NKX3-2 vector into SW1353 cells and FLAG-empty vector was used as a negative control. The next day, these cells were transfected with either a scrambled (Control RNAi) or NKX3-2 (NKX3-2 RNAi) siRNA duplex (100nM) and after 24 hours cells were harvested. Expression of mRNA for the indicated genes was determined by real-time RT-qPCR. Data were normalized to cyclophilin expression and set relative to the control condition (A-B: n=3 samples per condition). **C**. Translational capacity was determined and Puromycilation data were normalized to DNA content and calculated relative to the control condition (n=5 samples per condition). Statistical significance was determined using unpaired two-tailed Student's t-tests. Bars show the mean ±SEM. * $P<0.05$, ** $P<0.01$, *** $P<0.001$ versus control conditions. ns = not significant.

References

1. Caron MM, Emans PJ, Surtel DA, van der Kraan PM, van Rhijn LW, Welting TJ. BAPX-1/NKX-3.2 acts as a chondrocyte hypertrophy molecular switch in osteoarthritis. Arthritis Rheumatol. 2015;67(11):2944-56. Epub 2015/08/08. doi: 10.1002/art.39293. PubMed PMID: 26245691.
