# Supplementary material for: BMP7 increases protein synthesis in SW1353 cells and determines rRNA levels in a NKX3-2-dependent manner
Source: PLoS One. 2022 Feb 9;17(2):e0263430. doi: 10.1371/journal.pone.0263430 (PMC8827423; doi:10.1371/journal.pone.0263430)
Supplement: Attachment — Submitted filename: Response to Reviewers - Ripmeester et al..docx [file pone.0263430.s005.docx]

PONE-D-20-37112

BMP7 increases protein synthesis in SW1353 cells and determines rRNA levels in a NKX3-2-dependent manner

PLOS ONE

Dear Dr. Caron,

Thank you for submitting your manuscript to PLOS ONE. After careful consideration, we feel that it has merit but does not fully meet PLOS ONE’s publication criteria as it currently stands. Therefore, we invite you to submit a revised version of the manuscript that addresses the points raised during the review process.

We regret that it took a long time to evaluate your work, but it is not for lack of editorial effort. We consulted more than 30 experts (including suggested referees), and only recently we secured a second opinion. One of the reviewers is rather critical of your work, and we recommend that you address these comments to the extend possible by experimentation or otherwise modifications in the text. In the latter case, unresolvable concerns by reviewers as always could be adopted by you as your own acknowledgment of limitations to your work in the Discussion section. You could then take the opportunity to add how your work now adds to the field regardless of the current limitations, similar to the statements you would otherwise make in the rebuttal letter. The second reviewer was more enthusiastic about your work and provided constructive recommendations for revision that should not be insurmountable.

Please submit your revised manuscript by Mar 06 2021 11:59PM. If you will need more time than this to complete your revisions, please reply to this message or contact the journal office at plosone@plos.org. When you're ready to submit your revision, log on to https://www.editorialmanager.com/pone/ and select the 'Submissions Needing Revision' folder to locate your manuscript file.

Please include the following items when submitting your revised manuscript:

•	A rebuttal letter that responds to each point raised by the academic editor and reviewer(s). You should upload this letter as a separate file labeled 'Response to Reviewers'.

•	A marked-up copy of your manuscript that highlights changes made to the original version. You should upload this as a separate file labeled 'Revised Manuscript with Track Changes'.

•	An unmarked version of your revised paper without tracked changes. You should upload this as a separate file labeled 'Manuscript'.

If you would like to make changes to your financial disclosure, please include your updated statement in your cover letter. Guidelines for resubmitting your figure files are available below the reviewer comments at the end of this letter.

If applicable, we recommend that you deposit your laboratory protocols in protocols.io to enhance the reproducibility of your results. Protocols.io assigns your protocol its own identifier (DOI) so that it can be cited independently in the future. For instructions see: http://journals.plos.org/plosone/s/submission-guidelines#loc-laboratory-protocols

We look forward to receiving your revised manuscript.

Kind regards,

Andre van Wijnen

Academic Editor

PLOS ONE

Journal Requirements:

When submitting your revision, we need you to address these additional requirements.

1. Please ensure that your manuscript meets PLOS ONE's style requirements, including those for file naming. The PLOS ONE style templates can be found at

https://journals.plos.org/plosone/s/file?id=wjVg/PLOSOne_formatting_sample_main_body.pdf and

https://journals.plos.org/plosone/s/file?id=ba62/PLOSOne_formatting_sample_title_authors_affiliations.pdf

2.We note that you have a patent relating to material pertinent to this article. Please provide an amended statement of Competing Interests to declare this patent (with details including name and number), along with any other relevant declarations relating to employment, consultancy, patents, products in development or modified products etc. Please confirm that this does not alter your adherence to all PLOS ONE policies on sharing data and materials, as detailed online in our guide for authors http://journals.plos.org/plosone/s/competing-interests by including the following statement: "This does not alter our adherence to PLOS ONE policies on sharing data and materials.” If there are restrictions on sharing of data and/or materials, please state these. Please note that we cannot proceed with consideration of your article until this information has been declared.

This information should be included in your cover letter; we will change the online submission form on your behalf.

Please know it is PLOS ONE policy for corresponding authors to declare, on behalf of all authors, all potential competing interests for the purposes of transparency. PLOS defines a competing interest as anything that interferes with, or could reasonably be perceived as interfering with, the full and objective presentation, peer review, editorial decision-making, or publication of research or non-research articles submitted to one of the journals. Competing interests can be financial or non-financial, professional, or personal. Competing interests can arise in relationship to an organization or another person. Please follow this link to our website for more details on competing interests: http://journals.plos.org/plosone/s/competing-interests

- Authors’ response: We thank the Academic Editor for carefully reading our manuscript and the generous opportunity to revise our manuscript based on comments from the Academic Editor and the Reviewers*.*
- We amended the manuscript where necessary to meet PLOS ONE's style requirements and added the Competing Interest statement in the new Cover Letter.

Reviewers' comments:

Reviewer's Responses to Questions

Comments to the Author

1. Is the manuscript technically sound, and do the data support the conclusions?

The manuscript must describe a technically sound piece of scientific research with data that supports the conclusions. Experiments must have been conducted rigorously, with appropriate controls, replication, and sample sizes. The conclusions must be drawn appropriately based on the data presented.

Reviewer #1: Yes

Reviewer #2: Partly

________________________________________

2. Has the statistical analysis been performed appropriately and rigorously?

Reviewer #1: Yes

Reviewer #2: Yes

________________________________________

3. Have the authors made all data underlying the findings in their manuscript fully available?

The PLOS Data policy requires authors to make all data underlying the findings described in their manuscript fully available without restriction, with rare exception (please refer to the Data Availability Statement in the manuscript PDF file). The data should be provided as part of the manuscript or its supporting information, or deposited to a public repository. For example, in addition to summary statistics, the data points behind means, medians and variance measures should be available. If there are restrictions on publicly sharing data—e.g. participant privacy or use of data from a third party—those must be specified.

Reviewer #1: Yes

Reviewer #2: Yes

________________________________________

4. Is the manuscript presented in an intelligible fashion and written in standard English?

PLOS ONE does not copyedit accepted manuscripts, so the language in submitted articles must be clear, correct, and unambiguous. Any typographical or grammatical errors should be corrected at revision, so please note any specific errors here.

Reviewer #1: Yes

Reviewer #2: Yes

________________________________________

5. Review Comments to the Author

Please use the space provided to explain your answers to the questions above. You may also include additional comments for the author, including concerns about dual publication, research ethics, or publication ethics. (Please upload your review as an attachment if it exceeds 20,000 characters)

Reviewer #1: The authors have already published that BMP7 regulates rRNA levels via NKX3.2. Thus, the first two figures in this manuscript replicate their own previously published data, but using SW1353 cells rather than primary HACs. In addition, previously published studies have established a role for NKX3.2-mediated repression of RUNX2 in chondrogenic differentiation. Thus, this work fails to meet the journals requirement for novelty.

- Authors’ response: We thank the Reviewer for carefully reading our manuscript and we will address the remaining issues point-by-point below.
- The Reviewer is correct in stating that Figure 1A and 1B convey the same message as Figures published in the paper by Ripmeester *et al.* [1]. Furthermore, the link between BMP7-NKX3-2-RUNX2 repression axis was also previously reported by our group [2]. We also previously found that BMP7 can influence U3 snoRNA expression and thereby maturation of the 47S pre-rRNA [1]. However the involvement of BMP7 in 47S pre-rRNA transcription has not been shown before by us or other authors. In addition, the involvement of NKX3-2-RUNX2 repression as a potential mechanism of action for the observed BMP7 mediated increase in 47S pre-rRNA transcription was also not reported before. Indeed, this work builds upon our previous work but reports novel insights into the molecular interaction between BMP7 and chondrocyte ribosome biogenesis.

Additional issues:

Major: The siRNA studies in Figure 3 lack necessary specificity controls. These include; (1) the failure to use more than one NKX3-2 targeted siRNA duplex (2-3 distinct siRNAs should be used to reduce chance of off-target effects), (2) The authors should include a rescue experiment, by re-expression of a NKX3-2 mutant that evades riRNA silencing.

- Authors’ response: The siRNA against NKX3-2 in this manuscript is the same as the siRNA we previously published [2], were we showed more confirmation that knockdown of NKX3-2 by this siRNA resulted in specific effects on NKX3-2 target genes.

Due to the COVID19 crisis and subsequent restrictions in lab activities, we were not in the possibility to initiate new cell culture experiments plus all downstream activities that would be required for additional siRNA and NKX3-2 re-expression studies. We do however have a previously conducted experiment from which the data further harness that NKX3-2 is involved in regulating the expression of rRNAs. Overexpression of BAPX‐1/NKX3-2 by transfecting SW1353 cells with a validated 3xFLAG‐BAPX‐1/NKX3-2 vector [2] resulted in a significantly decreased expression of RUNX2 accompanied by an increased expression for 18S rRNA and 5.8S rRNA. These data are reciprocal to the NKX3-2 knockdown presented in Figure 3 and in line with the BMP7 mediated increase in rRNA expression from Figure 1. We hope that these data provide additional insight for this Reviewer on the role of NKX3-2 in chondrocyte rRNA expression. This Figure is now included in the Supplemental files accompanying this manuscript.

***S2 Fig: Overexpression of NKX3-2 results in increased 18S rRNA and 5.8S rRNA expression.*** *Overexpression of NKX3-2 was achieved by transfecting SW1353 cells with a validated 3xFLAG-NKX3-2 vector [2]. After 24 hours gene expression analysis was performed using RT-qPCR analysis. Data were normalized to cyclophilin expression and set relative to the empty-FLAG control condition (n=3 samples per condition). Increased FLAG-NKX3-2 expression was determined. Overexpression of FLAG-NKX3-2 resulted in decreased gene expression of RUNX2 and increased expression of 18S rRNA and 5.8S rRNA. 28S rRNA expression was not significantly changed by FLAG-NKX3-2. Statistical significance was determined using a two-tailed unpaired Student’s t-tests. Bars show the mean ±SEM. * P<0.05, ** P<0.01, *** P<0.001*.

Reviewer #2: The authors present a nicely written manuscript which investigates an interesting aspect in chondrogenic cell biology which not too many labs enquire. However, to fully prove the hypothesis presented, this work needs some further extension.

- Authors’ response: We thank the Reviewer for carefully reading our manuscript and emphasizing that research on protein translation in chondrocyte cell biology is not performed by too many labs. We are grateful for his/her positive remarks and we will happily address the remaining issues point-by-point below.

1. SUNsET assay: it is not very clear how this assay works in SW1353 cells. Please give some more details on how this assay reflects protein translation.

- Authors’ response: The SUNsET assay (surface sensing of translation) uses antibody detection of puromycin to monitor translation via immunohistochemistry. Puromycin mimics the terminus of aminoacyl-tRNA and hence is incorporated co-translationally in the growing nascent polypeptide chain. The amount of puromycin-incorporated peptides reflects the protein translational capacity of the cell, which can be determined by the intensity of the fluorescent signal [3].

2. To further prove the point of the paper it would be appropriate to show the expression of an unrelated/unaffected gene which is not controlled by the BMP7-NKX3-2- rRNA axis as a negative control.

- Authors’ response: We agree with the Reviewer that showing unrelated/unaffected gene expression by the BMP7-NKX3-2-rRNA axis would benefit the data presented in the manuscript as a negative control. The expression of the genes below are not significantly affected by either BMP7 or NKX3-2 knockdown, and this Figure is now included in the Supplemental files accompanying this manuscript.

***S1 Fig: Unrelated/unaffected genes which are not controlled by the BMP7-NKX3-2- rRNA axis. A/B****. SW1353 cells were transfected with either a scrambled (SCR) or NKX3-2 (NKX3-2 KD) siRNA duplex (100nM) and exposed to BMP7 (1nM) for 24 hours after which expression levels of Viperin, NFAT5, KRT18 or PTCH1 were determined using RT-qPCR analysis. Data were normalized to cyclophilin expression and set relative to the SCR control condition (n=3 samples per condition). Statistical significance was determined using a two-tailed unpaired Student’s t-tests, an no significant changes for each gene between conditions was observed. Bars show the mean ±SEM. * P<0.05, ** P<0.01, *** P<0.001.*

3. Next, the authors hypothesize that BMP7 influences the protein translational capacity in differentiating chondrocytes leading to a phenotypic change characterized by synthesis of the protein-rich part of the articular cartilage’s ECM. The authors need to demonstrate the direct link between the increased translation of proteins via ribosomal stimulation by BMP7 and to the articular cartilage ECM. Direct comparison between mRNA expression, protein expression and if possible protein ubiquitination of genes involved in cartilage ECM is necessary.

- Authors’ response: The Reviewer raises a valid point. In order to demonstrate that the effect of BMP7 on increased chondrocyte protein translation depends on rRNA transcription, we conducted an additional experiment. We treated SW1353 cells for 24 hours with Actinomycin D at 10 ng/ml, a concentration that selectively inhibits RNA polymerase I (PMID 21922053) [4]. RNA polymerase I is the only polymerase responsible for 47S rDNA transcription.

The data demonstrate that Actinomycin D mediated inhibition of rRNA transcription results in reduced chondrocyte protein synthesis. This could not be rescued by BMP7. These data strongly suggest that the effect of BMP7 on chondrocyte protein translation requires active rRNA transcription supporting our hypothesis. These data are now added to the main manuscript as Figure 1 C and D.

While we fully acknowledge the added value of measuring cartilage ECM specific protein synthesis in this context, we regret that we were not able to meet this request due to COVID19 related restrictions in local lab activities. We hope the Reviewer accepts this force majeure situation.

B

A

***A:*** *Translational capacity was determined using the SUNsET assay in SW1353 cells, which were exposed for 24 hours to Actinomycin D (10ng/ml) and BMP7 (1nM) or control conditions. Puromycilation data were normalized to DNA content and calculated relative to the control condition (n=5 samples per condition). Treatment with Actinomycin D reduced translational capacity of SW1353 cells, which could not be rescued by simultaneous exposure to BMP7.* ***B:*** *In similar samples as A, protein content was determined using a BCA assay. Treatment with Actinomycin D reduced total protein content, which could not be rescued by exposure to BMP7. Statistical significance was determined using unpaired Student’s t-tests. Bars show the mean (±SEM). ** P<0.01, *** P<0.001.*

4. A little graphical representation of the elucidated mechanism/pathway would add value to the manuscript and help all the readers which are not too familiar with control of rRNA's transcription and ribosomal translation.

- Authors’ response: We agree with the Reviewer that a graphical representation of the elucidated mechanism would benefit the manuscript. We thank the Reviewer for this helpful suggestion and included the graphical representation as shown below in the manuscript as Figure 4.

***Fig 4: Graphical representation of the elucidated mechanism.*** *The* *results of this study demonstrate that BMP7 increases protein translation and promotes rRNA transcription in SW1353 cells. rRNA transcription in SW1353 cells has been demonstrated to be mediated by a BMP7-induced inhibition of RUNX2 in a NKX3-2-dependent manner.*

**References:**

1.	Ripmeester EGJ, Caron MMJ, van den Akker GGH, Surtel DAM, Cremers A, Balaskas P, et al. Impaired chondrocyte U3 snoRNA expression in osteoarthritis impacts the chondrocyte protein translation apparatus. Scientific reports. 2020;10(1):13426-. doi: 10.1038/s41598-020-70453-9. PubMed PMID: 32778764.

2.	Caron MMJ, Emans PJ, Surtel DAM, van der Kraan PM, van Rhijn LW, Welting TJM. BAPX-1/NKX-3.2 Acts as a Chondrocyte Hypertrophy Molecular Switch in Osteoarthritis. Arthritis & Rheumatology. 2015;67(11):2944-56. doi: 10.1002/art.39293.

3.	Goodman CA, Hornberger TA. Measuring protein synthesis with SUnSET: a valid alternative to traditional techniques? Exerc Sport Sci Rev. 2013;41(2):107-15. Epub 2012/10/24. doi: 10.1097/JES.0b013e3182798a95. PubMed PMID: 23089927; PubMed Central PMCID: PMCPMC3951011.

4.	Bensaude O. Inhibiting eukaryotic transcription: Which compound to choose? How to evaluate its activity? Transcription. 2011;2(3):103-8. Epub 2011/09/17. doi: 10.4161/trns.2.3.16172. PubMed PMID: 21922053; PubMed Central PMCID: PMCPMC3173647.
